# Fluoroquinolone Resistance Mechanisms and population structure of *Enterobacter cloacae* non-susceptible to Ertapenem in North-Eastern France

**DOI:** 10.3389/fmicb.2015.01186

**Published:** 2015-10-23

**Authors:** Thomas Guillard, Pascal Cholley, Anne Limelette, Didier Hocquet, Lucie Matton, Christophe Guyeux, Anne-Laure Lebreil, Odile Bajolet, Lucien Brasme, Janick Madoux, Véronique Vernet-Garnier, Coralie Barbe, Xavier Bertrand, Christophe de Champs on behalf of CarbaFrEst Group

**Affiliations:** ^1^Laboratoire de Bactériologie-Virologie-Hygiène, Hôpital Robert Debré, CHU ReimsReims, France; ^2^Structure Fédérative de Recherche CAP-Santé, UFR Médecine, Université de Reims Champagne-ArdenneReims, France; ^3^Service d’Hygiène Hospitalière, CHRU BesançonBesançon, France; ^4^UMR 6249 Chrono-environnement, Université de Franche-ComtéBesançon, France; ^5^UMR 6174 CNRS, Département d’Informatique des Systèmes Complexes, Université de Franche-ComtéBelfort, France; ^6^Centre de Recherche et d’Investigation Clinique, Hôpital Robert Debré, CHU ReimsReims, France

**Keywords:** Fluoroquinolones, PMQR, QRDR, *Enterobacter cloacae*, Carbapenem, MLST

## Abstract

Fluoroquinolone (FQ) agents are a potential resort to treat infection due to *Enterobacteriaceae* producing extended spectrum β-lactamase and susceptible to FQ. In a context of increase of non-susceptibility to carbapenems among *Enterobacteriaceae*, we characterized FQ resistance mechanisms in 75 *Enterobacter cloacae* isolates non-susceptible to ertapenem in North-Eastern France in 2012 and describe the population structure by pulsed field gel electrophoresis (PFGE) and multi-locus sequence typing (MLST). Among them, 14.7% (12/75) carried a carbapenemase-encoding gene. Except one isolate producing VIM-1, the carbapenemase-producing isolates carried the well-known IncL/M pOXA48a plasmid. Most of the isolates (59/75) harbored at least a FQ-R determinant. *qnr* genes were predominant (40%, 30/75). The MLST study revealed that *E. cloacae* isolates’ clonality was wide [24 different sequence types (STs)]. The more widespread STs were ST74, ST101, ST110, ST114, and ST133. Carbapenem MICs were higher for *E. cloacae* ST74 than for other *E. cloacae* isolates. Plasmid-mediated quinolone resistance determinants were more often observed in *E. cloacae* ST74 isolates. These findings showed that (i) pOXA-48a is spreading in North-Eastern France, (ii) *qnr* is preponderant in *E. cloacae*, (iii) *E. cloacae* comprised a large amount of lineages spreading in North-Eastern France, and (iv) FQ as an alternative to β-lactams to treat ertapenem non-susceptible *Enterobacteriaceae* are compromised.

## Introduction

*Enterobacter cloacae* complex is, together with *Escherichia coli*, very common human *Enterobacteriaceae* pathogens. They can be a reservoir for infection due to their intestine colonization in patients upon long-term hospitalization and antimicrobial treatment. Due to the β-lactams antibiotic intensive use, rate of *Enterobacteriaceae* resistant to lactams-lactams highly increased these last years in French hospitals (Carbonne et al., 2013). Carbapenems are one of the last lines to treat patients infected with multidrug resistant *Enterobacteriaceae*. Thus, decreased susceptibility to carbapenems represents a threat of therapeutic dead ends. Two main mechanisms lead to decrease the susceptibility to carbapenems in *Enterobacteriaceae* isolates: (i) production of carbapenemase or (ii) production of other β-lactamase in combination with decreased permeability (Nordmann et al., 2011).

In France, OXA-48 is the most prevalent carbapenem-hydrolyzing β-lactamase and is a public health concern (Poirel et al., 2012b; Robert et al., 2014).

Recently in France, fluoroquinolone (FQ) agents have been proposed as the first choice to treat FQ-susceptible extended-spectrum β-lactamase-producing enterobacterial (ESBL) isolates in pyelonephritis in order to spare carbapenems^1^. Then, FQ can be used to treat infections due to carbapenem non-susceptible isolates. However, plasmid-mediated quinolone resistances (PMQR) confer a low level of quinolone resistance and they were shown to significantly reduce the activity of ciprofloxacin in urinary (Allou et al., 2009; Guillard et al., 2013) and respiratory (Rodríguez-Martínez et al., 2008; Dominguez-Herrera et al., 2013) tract infection murine models. Multi-locus sequence typing (MLST) has been widely used to study population structure of *E. coli* and *K. pneumoniae*. Although MLST has also been described for *E. cloacae*, only few studies are available (Miyoshi-Akiyama et al., 2013; Izdebski et al., 2014).

The aim of this study was to characterize FQ resistance determinants carried by *E. cloacae* isolates showing a non-susceptibility to carbapenems and to assess the population structure of these isolates in order to better understand their spread in French hospitals.

## Materials and Methods

### Selection of Bacterial Strains

The survey was conducted in the five teaching hospitals (UH1: Besançon, UH2: Dijon, UH3: Nancy, UH4: Reims, and UH5: Strasbourg) and two general hospitals (GH1: Colmar and GH2: Troyes) in North-Eastern France from January 1st to June 30th, 2012. Medical data settings of the seven hospitals together were 10,311 beds and almost 540,000 admissions per year all through the period of investigation. After detection on chromID^®^ ESBL agar plates (bioMérieux, Marcy l’Etoile, France), all non-duplicate *E. cloacae* isolates from clinical or screening samples non-susceptible to ertapenem (MIC ertapenem >0.5 μg/mL) were collected and sent to Reims Hospital laboratory.

### Identification, Susceptibility Testing, and ESBL Detection

Identification of isolates was performed using MALDI-TOF (Bruker Daltonics, Bremen, Germany). Antibiotic susceptibilities were determined by the disk diffusion method according to European Committee on Antimicrobial Susceptibility Testing (EUCAST) guidelines^2^ and ESBLs were detected by the double-disk synergy test as previously described (Jarlier et al., 1988; Duval et al., 2009). AmpC overexpression was detected by disk diffusion method performed on Mueller-Hinton agar supplemented with cloxacillin (250 μg/mL). Carbapenem (imipenem, ertapenem, doripenem, and meropenem) and FQ (norfloxacin, ofloxacin, nalidixic acid, and ciprofloxacin) MICs were determined using *E* test^®^ strips (bioMérieux, Marcy l’Etoile, France) according to the manufacturer’s recommendations and interpreted according to EUCAST. Metallo-β-lactamase production was screened with the MβL *E* test (bioMérieux, Marcy l’Etoile, France). In addition, metallo-β-lactamase was detected with imipenem disk with and without EDTA (5 μL of 0.5 M EDTA per disk) as previously described (Liapis et al., 2014).

### β-Lactamase Detection

Carbapenemase-encoding genes *bla*_KPC_, *bla*_V IM_, *bla*_IMP_, *bla*_NDM_, *bla*_OXA-23_-like, *bla*_OXA-24_-like, *bla*_OXA-58_-like, and *bla*_OXA-48_-like were screened using multiplex PCRs and completed with simplex PCRs for *bla*_IMI_ and *bla*_GES_ as described elsewhere (Gharout-Sait et al., 2014). All the *bla*_OXA-48_-like detected were subsequently sequenced. Genes *bla*_TEM_, *bla*_SHV_, *bla*_CTX-M_, and *bla*_OXA_ were detected by PCR using specific primers and sequenced as previously described for all the ESBL-producing strains (Guillard et al., 2014a). Plasmid-mediated AmpC-type genes *bla*_ACC_, *bla*_FOX_, *bla*_MOX_, *bla*_DHA_, *bla*_CMY_, and *bla*_MIR_ were screened using multiplex PCRs and sequenced as previously described (Gharout-Sait et al., 2014).

### Quinolone Resistance Determining Region (QRDR) and PMQR Identification

The quinolone resistance determining region (QRDR) was amplified by PCR and sequenced in the *gyrA*, *gyrB*, *parC*, and *parE* genes, as described elsewhere (de Lastours et al., 2012). *qnr*, *qepA*, and *oqxAB* genes were detected by real-time PCR, *aac(6’)-Ib-cr* was detected by pyrosequencing as described elsewhere (Guillard et al., 2010, 2011, 2015).

### Contribution of Eﬄux and Reduced Outer-membrane Permeability

For contribution of eﬄux and reduced outer-membrane permeability study, we randomly selected five strains that did not produce OXA-48 and three, which produced OXA-48.

Quantitative real-time reverse transcriptase PCR (qRT-PCR) was performed to determine the expression of *ompF* and *ompC* porin genes and the *acrB* eﬄux pump gene, relative to the *rpoB* housekeeping gene. From bacteria grown to mid-exponential growth phase in LB, total RNA was extracted using RNeasy Kit (Qiagen, Courtaboeuf, France) as recommended by the manufacturer. Residual chromosomal DNA was removed by treating samples with the RNAase free DNase I (Qiagen) and TURBO DNA-free kit (Life Technologies). DNase-treated RNA samples were quantified using the easy-to-use Qubit^TM^ 3.0 Fluorometer (ThermoFisher Scientific). qRT-PCR experiments were performed using the KAPA SYBR^®^ FAST One-Step qRT-PCR Kit Universal (Kapa Biosystems, USA) and the LightCycler 480 (Roche Molecular Diagnostics, Germany) according to manufacturers’ recommendations. Each experiment was performed in triplicate. RNA transcript levels were calculated using the 2^-ΔΔCt^ method following the MIQE checklist (Bustin et al., 2009), and are expressed relative to levels in the *E. cloacae* CIP 60.85T control. Primers used are presented in Supplementary Table S1.

Bacterial outer-membrane proteins (OMPs) were detected by SDS/PAGE as previously described (Martínez-Martínez et al., 2002). Briefly, bacterial cells were broken by sonication, and membranes were collected by ultracentrifugation at 100,000 × *g* for 1 h. The inner membrane was solubilized with 1% sodium *N*-lauroylsarcosinate. Proteins in the outer membrane were separated by SDS/PAGE and gels were stained with Coomassie blue for visualization.

### Plasmids Characterization

Plasmids carrying *bla*_OXA-48_ were studied as described elsewhere (Guillard et al., 2014b). Briefly, plasmid extraction was performed by the Kieser method. Transfer of the plasmids was then studied by conjugation assays using azide-resistant *E. coli* J53 as a recipient cell and counter-selection with ertapenem 0.5 μg/mL, rifampicin 250 μg/mL, and sodium azide 100 μg/mL. Incompatibility groups of the plasmids were eventually determined using the PCR-based replicon typing (PBRT) for the paired strains and transconjugants (Carattoli et al., 2005). The incL/M group was characterized using the primers described by Poirel et al. (2012a). Genetic environment of *bla*_OXA-48_ was studied by PCR mapping to seek for an environment such as *Tn1999.2* using primers listed in Supplementary Table S1.

### Genotyping

Pulsed-field gel electrophoresis (PFGE) was performed on *E. cloacae* isolates using the *Xba*I restriction enzyme as previously described and results were interpreted according to international criteria (Tenover et al., 1995).

### Analysis of MLST Data

Multi-locus sequence typing was performed according to published protocols (Diancourt et al., 2005; Miyoshi-Akiyama et al., 2013). Clonal complexes (CC) were defined as a group of STs sharing at least five loci. In order to build a dendrogram with the 373 sequence types (STs) available at the time of the study (including the new ST described in this collection), we concatenated the sequences of seven MLST genes to form a 3,511-bp sequences alignment, defining 914 polymorphic positions. The best-fit nucleotide substitution model for this data was GTR + G + I, as determined with jModelTest 0.1.1. We used the *Enterobacter aerogenes* KCTC2190 as the outgroup strain. Maximum likelihood tree was constructed with RAxML 7.2.8 and visualized with Dendroscope 3.2.10 (Stamatakis, 2006; Huson and Scornavacca, 2012). In every case, 1000 bootstrap repetitions gave values above 900 for most branches.

### Statistical Analysis

Qualitative variables were analyzed with the Chi2 test and the two-tailed Fisher exact test. Quantitative variables were compared using the Mann–Whitney test. The results were considered statistically significant when *P* < 0.05.

## Results

### Species Distribution of the Ertapenem Non-susceptible *Enterobacteriaceae* Isolates

The 75 *E. cloacae* isolates were distributed as follow in the different centres: 21 (28.0%) in UH4, 18 (24.0%) in UH3, 13 (17.3%) in GH2, 10 (13.3%) in UH5, 9 (12.0%) in UH1, 3 (4.0%) in UH2, and 1 (1.3%) in GH1. They were isolated from clinical samples (65.3%; *n* = 49: 22 (29.3%) urines, 5 (6.7%) blood, 22 (29.3 %) other infectious sites) or from 26 screening samples (34.7%; stools or rectal swabs). The global prevalence of carbapenem-non-susceptible isolates in *E. cloacae* species was 6.05% (0.91–8.36 according to the centre).

### Antibiotic Susceptibility and Level of Resistance

All isolates but three were intermediate or resistant to piperacillin and all but one to ticarcillin–clavulanic acid. Ten point seven percent were susceptible to piperacillin–tazobactam, 4.0% to aztreonam, 6.7% to cefotaxime, 22.7% to cefpirome, 46.7 to cefepime, 33.3% to tobramycin, 38.7% to gentamicin, 96.0% to amikacin, 52.7% to tetracycline, 45.9% to chloramphenicol, 17.3% to nalidixic acid, 18.7% to ofloxacin, 26.7% to ciprofloxacin, 18.7% to norfloxacin, 57.3% to co-trimoxazole, 92.0% to fosfomycin, 93.3% to imipenem, 97.3% to meropenem, and 96.0% to doripenem. The frequency of isolates susceptible to FQ agents among clinical isolates did not differ from that of screening isolates (**Table 1**).

**Table 1:** Fluoroquinolones MIC (μg/mL) among the clinical and the screening *Enterobacter cloacae* isolates.

		Nalidixic acid			Norfloxacin			Ciprofloxacin			Ofloxacin		
	*n*	MIC_50_	MIC_90_	Range	MIC_50_	MIC_90_	Range	MIC_50_	MIC_90_	Range	MIC_50_	MIC_90_	Range
Screening isolates	26	>256	>256	3->256	>256	>256	0,125–>256	>32	>32	0,016–>32	> 32	>32	0,125–>32
Clinical isolates	49	>256	>256	0,19->256	12	>256	0,064–>256	2	>32	0,012–>32	8	>32	0,094–>32
Urines	22	>256	>256	2->256	9	>256	0,064–>256	2	>32	0,012–>32	6	>32	0,094–>32

### β-Lactam Resistance Determinants

Carbapenemases were not the major determinant leading to decrease susceptibility to carbapenems in *E. cloacae* isolates. Indeed, among them, 84% (63/75) did not produce a carbapenemase. Nonetheless, among the carbapenemase-producing isolates, OXA-48 (11/12 for *E. cloacae*) was the most prevalent carbapenemase, and interestingly the majority of OXA-48-producing isolates co-harbored CTX-M-15 and TEM-1 (11/11). One isolate produced VIM-1.

Focusing on isolates, which did not produce a carbapenemase, most of them (40/63) did not produce ESBL, and 29 out of 63 did not produce any acquired β-lactamases. These strains did not carry any plasmid-mediated AmpC-type genes except one harboring *bla*_DHA-1_. The eight strains, studied for eﬄux and porins expression, produced an overexpressed AmpC except one. This latter did not produce any acquired β-lactamases. Ertapenem MICs > 3 mg/L were related to a decreased expression of *ompF* and *ompC*, and for three strains with a lost a porin (**Table 2**).

**Table 2:** Contribution of eﬄux and reduced outer membrane permeability to carbapenems for *E. cloacae* strains resistant to ertapenem.

Strain	ST	β-lactamases	MIC (μg/mL)				SDS PAGE	Relative expression^a^		
			Ertapenem	Imipenem	Doripenem	Meropenem		*ompF*	*ompC*	*acrB*
12 01 033	133	SHV-12, CTX-M-9, overexpressed AmpC	>32	16	4	6	Loss of OmpC	0.22	0.02	0.51
12 01 040	133	SHV-12, CTX-M-9, overexpressed AmpC	>32	12	4	12	Loss of OmpC	0.23	0.03	0.7
12 05 006	175	SHV-12, overexpressed AmpC	32	2	0.75	1	Loss of OmpF	0.05	0.11	2.28
12 03 029	74	CTX-M-15	2	2	0.5	2	–	0.44	0.22	2.76
12 06 020	102	–	1.5	2	1.5	1.5	–	0.52	0.36	2.12
12 05 004	74	OXA-48, CTX-M-15	6	1.5	0.75	1.5	–	3.15	0.54	9
12 03 022	110	OXA-48, CTX-M-15	3	1	0.75	0.5	Modified porins	3.23	0.009	6.57
12 03 028	74	OXA-48, CTX-M-15	2	1	0.25	0.75	-	0.44	0.22	2.76

*^a^Relative expression is calculated as –2^ΔΔCt^, where ΔΔCt = (Ct_target_ – Ct_reference_). The targets were each strain studied and the reference was the reference strain E. cloacae CIP 60.85T.*

All the strains co-producing OXA-48 and CTX-M-15 were susceptible to carbapenems but ertapenem, with MICs close to the breakpoints. The three OXA-48 and CTX-M-15-producing strains, selected for eﬄux and outer membrane study, showed an *acrB* overexpression of at least a twofold change (**Table 2**). All these strains showed also a decreased *ompC* expression.

### *bla*_OXA-48_ is Carried by an IncL/M Conjugative Plasmid

To study plasmids carrying *bla*_OXA-48_, we selected three strains to be studied among the *E. cloacae* OXA-48 producing-isolates according to antibiotic resistance genes characterization. Mating out assays and plasmid DNA analysis allowed identification of a 62-kb-long plasmid carrying *bla*_OXA-48_ but not *bla*_TEM-1_ and *bla*_CTX-M-15_. This conjugative plasmid belonged to the incL/M incompatibility group. Mapping of the genetic environment showed that *bla*_OXA-48_ was embedded in *Tn1999*.2 as previously described (Poirel et al., 2004). As expected, OXA-48-producing transconjugants showed a low level of resistance to carbapenems (data not shown).

### FQ Resistance Determinants

Most of the *E. cloacae* (59/75, 78.7%) isolates harbored a FQ-R determinant. *E. cloacae* isolates carried in the same proportion either substitutions in QRDR only (37%, 28/75) or association of PMQR and substitutions in QRDR (36%, 27/75). Very few isolates produced PMQR solely (5%).

Focusing on PMQR harbored by the *E. cloacae* isolates, we showed that only *qnr* genes and *aac(6’)-Ib-cr* were detected. *qnr* genes were predominant (40%, 30/75) compared to *aac(6’)-Ib-cr* (23%, 17/75), (*P* < 0.02). Three *qnr* families were found (*qnrA*, *qnrB*, and *qnrS*) with a majority of *qnrB* (18/30). Looking more carefully at the alleles, we found 9 *qnrA1*, 16 *qnrB1*, 2 *qnrB2* and 3 *qnrS1.* Of the 31 PMQR-harboring *E. cloacae* isolates, 13 (42%) carried *qnr* only and 17 (55%) *qnr* associated with *aac(6’)-Ib-cr*. One was AAC(6’)-Ib-cr producer only.

Substitutions in the QRDR were detected at high rate. Our findings showed that 73% (55/75) of isolates carried substitutions in QRDR. Majority of isolates (51%, 38/75) harbored substitutions in GyrA only. More precisely, *E. cloacae* isolates carried predominantly either one substitution in GyrA (48%, 36/75) or a combination of two substitutions in GyrA and one substitution in ParC (18.7%, 14/75). The GyrA substitutions Ser/Thr83Phe and Ser/Thr83Ile (49 and 36%, respectively) were the most frequent (**Table 3**). No substitutions were characterized in *gyrB* and *parE*.

**Table 3:** Mutations observed in QRDR of *E. cloacae* isolates.

Amino acid substitutions			No. of isolates (Total of 75)
GyrA		ParC	
Ser/Thr83	Asn87	Ser80	
–	–	–	20
Ile	–	–	19
Ile	–	Ile	1
Phe	–	–	11
Phe	–	Ile	1
Phe	Asn	–	2
Phe	Asn	Ile	10
Phe	Gly	Ile	3
Leu	–	Ile	1
Tyr	–	–	6
Tyr	His	Ile	1

*(–) Indicates no mutation found at the location.*

As shown in **Table 4**, 82.6% of PMQR-producing *E. cloacae* had substitution in QRDR vs. 60.0% in PMQR-negative. But the number of substitutions in QRDR was higher in PMQR-negative isolates (e.g., 3 mutations: 32.5% vs. 0% for PMQR-negative and PMQR-producing isolates, respectively, *P* < 0.0001). For this analysis, we deleted duplicates in each PFGE if they carried same PMQR associated to same mutations pattern in QRDR.

**Table 4:** Prevalence of QRDR mutations in PMQR-carrying and PMQR negative *E. cloacae* isolates^a^.

*E. cloacae*	PMQR+		PMQR–	
QRDR+	19	82.6%	24	60.0%
QRDR–	4	16.4%	16	40.0%
0 mutation	4	17.4%	16	40.0%
1 mutation	17	74.0%	9	22.5%
2 mutations	2	8.6%	2	5.0%
3 mutations	0	0.0%	13	32.5%
**Total**	23		40	

*^a^After exclusion of duplicates: one isolate was included in each PFGE harboring different PMQR and mutations pattern in QRDR compared to the other isolates included in the same PFGE profile.*

### Epidemiological Links of *E. cloacae*

**FIGURE 1:**
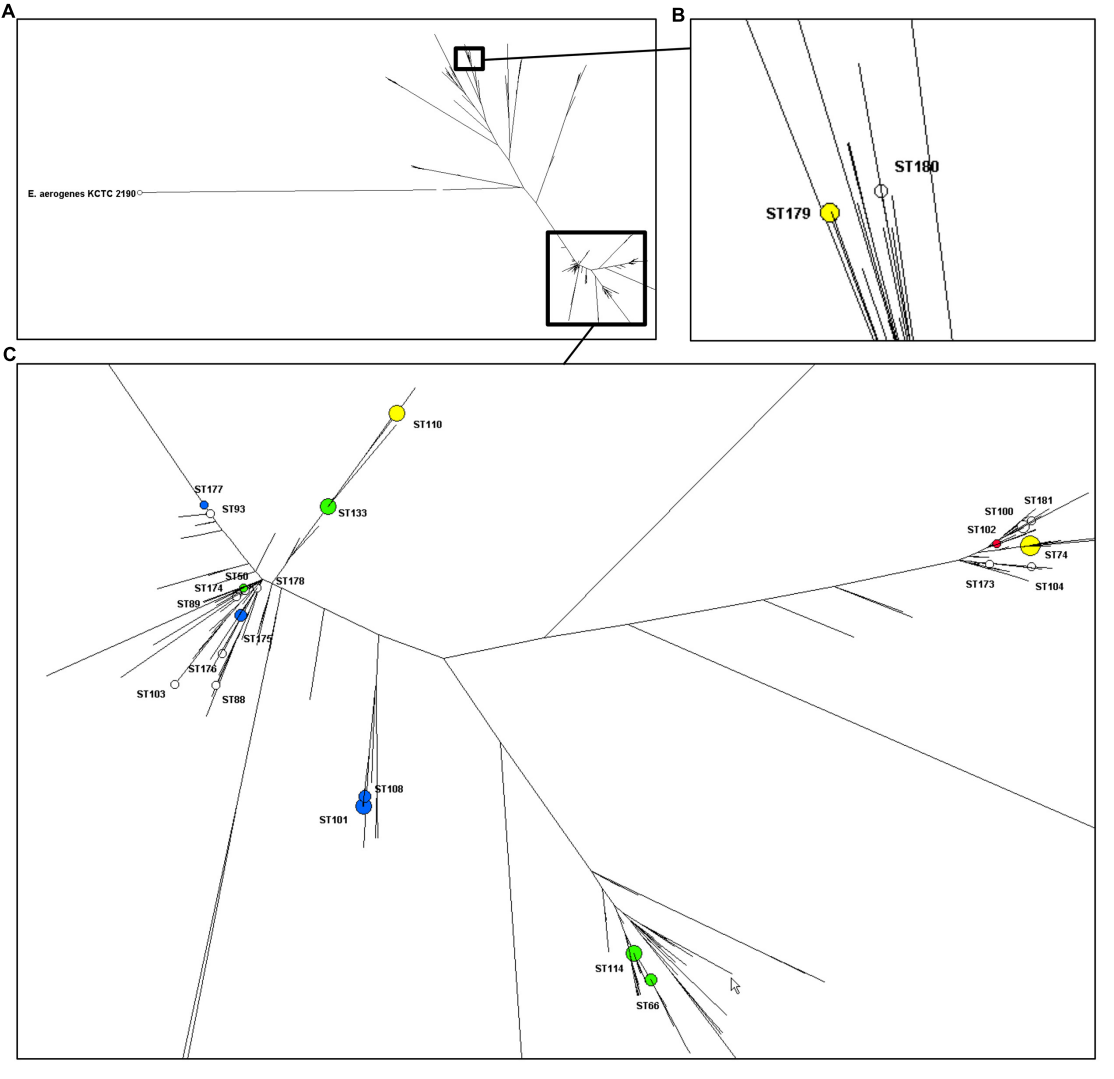
**Distribution of the STs of 75 clinical isolates of *Enterobacter cloacae* (non-susceptible to carbapenems and isolated in North-Eastern France) on a dendrogram built with the data all known STs (*n* = 373). (A)** Overview of the dendrogram with *E. aerogenes* as outgroup. **(B)** Close-up of the branch with ST179 and ST180. **(C)** Close-up the branch harboring the rest of the isolates described in this study. Enzymes in the different ST are represented with blue (SHV/TEM), yellow (OXA-48), green (CTX-M), and red (VIM) spots. Spots’ size depends on the amount of strains.

Typing results of *E. cloacae* isolates are shown in **Table 5**. Seventy-four isolates (one was untypeable with PFGE) were distributed into 40 pulsotypes and the 75 isolates clustered into 24 STs of which 12 were grouped into five CCs (CC42, CC74, CC114, CC133, and CC234). ST74 was the most frequent ST accounting for 16 out of 75 isolates (21%), followed by ST101 (nine isolates, 12%) ST110 and ST133 (eight isolates each, 11%) and ST114 (six isolates, 8%). Eight STs were split into two to five pulsotypes and the two most predominant STs, ST74 and ST101, were also the most diverse. The phylogenetic tree built from the sequence data of the seven MLST genes of the 373 STs of *E. cloacae* clearly showed that the great majority of our isolates clustered in one phylogenetic group (**Figure 1**).

**Table 5:** *E. cloacae* STs identified in our study: clonal status, pulsotypes, centres, prevalence, carbapenem MIC and β-lactamases.

CC (ST)^a^	PFGE profiles	Centres	Isolates (*n*)	Median MIC (range; μg/mL)								β-lactamases (number of isolates)^b,c^


				Imipenem		Ertapenem		Doripenem		Meropenem		
42 (101, 104, 108)	2,8,10,14,17–20	UH1, UH3, UH4, UH5, GH1, GH2	13	0.38	(0.13–1)	1	(0.75–4)	0.125	(0.047–0.75)	0.125	(0.064–0.5)	SHV-12 (*n* = 1)
74 (74)	31–33,35,41	UH2, UH3, UH4, UH5, GH2,	16	0.62	(0.13–2)	2	(0.75–12)	0.25	(0.064–1)	0.44	(0.064–2)	OXA-48 (*n* = 9), CTX-M-15 (*n* = 12)
114 (66,114)	3,5,40	UH1, UH3, UH5	7	0.38	(0.13–2)	2	(1–6)	0.125	(0.064–0.38)	0.19	(0.125–0.5)	CTX-M-9 (*n* = 1) CTX-M-15 (*n* = 3), TEM-24 (*n* = 1), SHV-12 (*n* = 1)
133 (93, 103,133)	15,21,23,38,39	UH3, UH4, UH5,	10	0.44	(0.19–16)	2	(0.75–32)	0.125	(0.16–4)	0.16	(0.016–12)	CTX-M-9 (*n* = 7), SHV-12 (*n* = 3)
234 (50,176,178)	24,34,37	UH2, UH3	3	0.5	(0.38–1)	2	(0.75–12)	0.25	(0.064–0.5)	0.38	(0.064–0.5)	CTX-M-9 (*n* = 1), SHV-12 (*n* = 1)
ST110	28,29,30	UH1, UH4, GH2	8	0.38	(0.25–1)	1.25	(0.75–3)	0.125	(0.064–0.75)	0.16	(0.047–0.5)	OXA-48 (*n* = 1), CTX-M-15 (*n* = 1), SHV-12 (*n* = 2)
ST179	ND,26,27	UH3, GH2	4	0.5	(0.5–0.75)	1	(0.75–4)	0.094	(0.064–0.38)	0.094	(0.064–0.75)	OXA-48 (*n* = 1), CTX-M-15 (*n* = 1)
ST100	6,7	UH4	2	0.25	(0.25–0.25)	1.25	(1–1.5)	0.063	(0.032–0.094)	0.063	(0.032–0.094)	–
ST175	16	UH1, UH2	2	1.125	(0.25–2)	16.25	(0.75–32)	0.47	(0.19–0.75)	0.56	(0.125–1)	SHV-12 (*n* = 1), DHA-1 (*n* = 1)
ST88,89, 102,173, 174,177, 180,181^b^	1,4,9,11–13, 25,36	UH1, UH4, UH5	10	0.25	(0.13–1)	0.88	(0.75–32)	0.1	(0.047–0.75)	0.079	(0.064–2)	CTX-M-15 (*n* = 1), SHV-12 (*n* = 2), VIM-1 (*n* = 1)

*^a^For E. cloacae, clonal complex determined according to Izdebski et al. (2014).*

*^b^Carbapenemase, extended spectrum β-lactamase, plasmid-mediated cephalosporinase. TEM-1 and TEM-2 have not been mentioned.*

*^c^One isolate in each ST.*

*ND, Not determined; NT, Non-typeable.*

*Enterobacter cloacae* ST74 isolates were more often less susceptible than the other *E. cloacae* isolates to various antimicrobial agents (**Table 6**). They included nine of the 11 OXA-48 producing isolates observed.

**Table 6:** Susceptibility rates of *E. cloacae* isolates to various antimicrobials according to the ST type.

	No. of susceptible isolates (%)				
Antimicrobial agent	ST74		Other ST		*P*-value
	*n*	(%)	*n*	(%)	
Ticarcillin + Clavulanic acid	0	(0.0)	1	(1.7)	NS
Piperacillin + Tazobactam	2	(12.5)	6	(10.2)	NS
Aztreonam	0	(0.0)	3	(5.1)	NS
Cefotaxime	2	(12.5)	3	(5.1)	NS
Ceftazidime	2	(12.5)	2	(3.4)	NS
Cefepime	4	(25.0)	31	(52.5)	0.05
Cefpirome	3	(18.8)	14	(23.7)	NS
Ertapenem	0	(0.0)	0	(0.0)	NS
Imipenem	15	(93.8)	55	(93.2)	NS
Meropenem	16	(100.0)	57	(96.6)	NS
Doripenem	16	(100.0)	56	(94.9)	NS
Tobramycin	2	(12.5)	23	(39.0)	0.04
Gentamicin	3	(18.8)	26	(44.1)	NS (0.06)
Amikacin	14	(87.5)	58	(98.3)	NS
Tetracycline	10	(62.5)	29	(50.0)	NS
Chloramphenicol	3	(18.8)	31	(53.4)	0.05
Nalidixic acid	0	(0.0)	13	(22.0)	NS
Ofloxacin	1	(6.3)	13	(22.0)	NS
Ciprofloxacin	1	(6.3)	19	(32.2)	0.05
Co-trimoxazole	2	(12.5)	41	(69.5)	<0.001
Fosfomycin	15	(93.8)	54	(93.1)	NS


Plasmid-mediated quinolone resistances determinants were more frequent in *E. cloacae* ST74 isolates than in non-ST74 (87% vs. 40% *P* = 0.001). 81% (13/16) of ST74 isolates harbored *qnr* vs. 29 % (17/58) of non-ST74 (*P* = 0.0002). Seventy-five percent (12/16) of ST74 isolates contained *aac(6’)-Ib-cr* vs. 9% (5/58) of non-ST74 (*P* = 4.10^-7^).

Ten of the 16 ST74 isolates were observed in GH2. They shared the same pulsotype 32, produced the identical β-lactamases and PMQR (CTX-M-15, TEM-1, QnrB1, and AAC(6’)-Ib-cr) and 8 of them produced OXA-48. It appears that the spread of *bla*_OXA-48_ in *E. cloacae* was mainly due to an epidemic strain.

## Discussion

Although FQ use has been reported recently as a risk factor for infections due to carbapenem-resistant *K. pneumoniae* (Hussein et al., 2009), French guidelines for urinary tract infection treatment stated that FQ remains the first line treatment for pyelonephritis due to non-producing or producing-ESBL but FQ-susceptible enterobacterial isolates. These guidelines should help to decrease unnecessary carbapenem use to treat ESBL-producing enterobacterial isolates. So, we decided to study FQ resistance mechanisms in *Enterobacteriaceae* isolates non-susceptible to carbapenems in North-Eastern France: (i) we determined carbapenem susceptibility and β-lactamase detection; (ii) we characterized PMQR and mutations in QRDR for *E. cloacae*; and (iii) we defined the population structure of *E. cloacae* isolates.

### β-Lactam Resistance Determinants

Carbapenemases were not the major determinant leading to decrease susceptibility to carbapenems in *E. cloacae* isolates. Nonetheless, OXA-48 was the most prevalent carbapenamase. All the strains co-harboring OXA-48 and CTX-M-15 were susceptible to carbapenems but ertapenem, with MICs close to the breakpoints. But interestingly, contribution of eﬄux and/or reduced outer membrane permeability was important in those strains. Reports about carbapenem non-susceptibility of *E. cloacae* were scarce and OXA-48-producing *E. cloacae* were rarely reported (Hammoudi et al., 2014; Majewski et al., 2014; Pantel et al., 2014). Then, the amount of OXA-48 *E. cloacae* isolates in our study showed increase of this carbapenemase in this species in France. In opposite to Hammoudi et al. (2014) report in Lebanon, carbapenemase production is not the major cause of carbapenem non-susceptibility in our isolates. The part played by ESBL or AmpC was previously reported and DHA-1 has been shown to reduce imipenem activity combined with loss of porins (Mammeri et al., 2010). This latter point is underscored by our results showing combination of overexpressed eﬄux and outer membrane impermeability for all the strains we sampled.

### FQ Resistance Determinants

To our knowledge, few epidemiological studies have recently reported data about PMQR and/or substitutions in QRDR in *E. cloacae*, while huge data are available for *E. coli*. Majority of the *E. cloacae* isolates harbored a FQ-R determinant. *E. cloacae* isolates carried in the same proportion either substitutions in QRDR only (37%), or association of PMQR and substitutions in QRDR (36%). Very few isolates produced PMQR solely (5%). These findings are different from those reported by Ferjani et al. (2014) stating that *E. cloacae* strains, isolated in 2010 in Tunisia, carried predominantly *qnr* genes (50%) but less substitutions in QRDR (50%). Recently, it has been shown that *E. cloacae* non-susceptible to carbapenem isolated in China from 2009 to 2012 were highly PMQR carriers (68.9%), but no data about QRDR were available (Huang et al., 2012). Our results about eﬄux and outer membrane permeability confirmed that reduced outer membrane permeability and increased eﬄux are an important mechanism of FQ resistance in *E. cloacae*, as previously reported for this specie (Davin-Regli and Pagès, 2015; Li et al., 2015). Moreover as suggested by Davin-Regli and Pagès (2015) this particular property could be an asset for spreading in hospitals. We did not test all the strains for impermeability but regarding of the results we obtained from the sampled strains, overexpression of eﬄux pumps might be consider as a major resistance determinant in *E. cloacae* (Davin-Regli and Pagès, 2015). It has been proposed that OmpC disappears before OmpF in *E. aerogenes* in the presence of selective pressure from antibiotics and could be extend to other *Enterobacteriaceae* {de Champs et al., 1993ce, Lavigne et al., 2013wz}. It is noteworthy that our results are in agreement with this statement except for one strain, which lacked OmpF and had a decreased expression of OmpC. Similar results have been also reported for *E. cloacae* without any clear information about this discrepancy {Doumith et al., 2009ks}.

### *qnr* is the Major PMQR Determinant in *E. cloacae*

Focusing on PMQR harbored by the *E. cloacae* isolates, we showed that only *qnr* genes and *aac(6’)-Ib-cr* were detected. *qnr* genes were predominant (40%, 30/75) and *qnrB* was the most predominant family.

In a study conducted on ESBL-producing *Enterobacteriaceae* in Tunisia, it has been shown that *qnr* genes were mainly detected in *E. cloacae* (50%) and *aac(6’)-Ib-cr* genes in *E. coli* (47.5%) isolates (Ferjani et al., 2014). Huang et al. studied the prevalence of PMQR among carbapenem non-susceptible *E. cloacae* isolated between 2009 and 2012 in China (Huang et al., 2012). From the 986 isolates, 35 (3.55%) were non-susceptible to carbapenem. In their study, *qnr* genes were detected in 75% of the isolates whereas only 15% carried *aac(6’)-Ib-cr*.

### *E. cloacae* Isolates have a Wide Range of Substitutions Patterns

Substitutions in the QRDR were detected at high rate in the *E. cloacae* isolates (73%) studied here. Majority of isolates (51%) harbored substitutions in GyrA only, with Ser/Thr83Phe and Ser/Thr83Ile (49 and 36%, respectively) as the most frequent. These results differed from those previously reported for *E. coli*, stating that the GyrA substitution Ser83Leu is the most prevalent substitution in QRDR (Fendukly et al., 2003). As no substitutions were characterized in *gyrB* and *parE*, our findings underscored that *gyrB* and *parE* substitutions in *E. cloacae* are very rare. Indeed, these results were mostly described in *E. coli* isolates (Uchida et al., 2010), but our findings suggest a similar trend for *E. cloacae.*

GyrA substitutions were observed predominantly as single substitution in QRDR in *E. cloacae*, but ParC mutations were always combined with GyrA substitution. These results are in agreement with others studies and with the hypothesis that, in *Enterobacteriaceae*, DNA gyrase is the primary target enzyme where substitutions occur to resist against FQ. Topoisomerase IV, described as less sensitive to FQ than DNA gyrase, is the secondary target (Hooper, 2001). First-step quinolone resistance mutations occur in *gyrA* and mutations in *parC* occurred as a secondary event leading to highly resistant *Enterobacteriaceae* isolates.

The aims of our study were not to determine contribution of QRDR substitution to resistance, but it is well known that substitutions in hot spot of QRDR lead to high level of resistance as observed in this study (Hooper, 2001).

### PMQR-negative *E. cloacae* Isolates Harbor Multiple Substitutions in QRDR

Impact of PMQR on substitution in QRDR under FQ pressure is still debating. Of course, in literature this issue was mainly studied for *qnr*. In one hand, it has been largely reported that Qnr proteins increase rate of substitution in QRDR by increasing the mutant selection concentration. But, in the other hand, it has been also observed in *E. cloacae* that fewer QRDR substitutions occurred in Qnr-producing isolates (Lascols et al., 2007). That was consistent with molecular analysis showing that Qnr protects QRDR, in gyrase and Topoisomerase IV, from substitution under FQ inhibition (Cesaro et al., 2008). So, we looked eventually at the prevalence of QRDR substitutions in PMQR-containing and PMQR-negative *E. cloacae* isolates trying to gain insight how QRDR substitutions are affected by presence of PMQR. Our findings seem to support the hypothesis that presence of PMQR may confer a low-level resistance promoting at least one substitution in QRDR. Although, harboring no PMQR seems to lead to multiple substitutions in QRDR. We could hypothesize that a strain carrying no PMQR may acquire one chromosomal substitution in GyrA under FQ pressure. Then, if the strain carries at least Qnr, this latter may avoid multiple substitutions by protecting QRDR domains.

### Epidemiological Links of *E. cloacae*

Typing results of *E. cloacae* isolates showed ST74 as the most frequent ST with the lowest susceptibility against β-lactams and carrying PMQR frequently.

To our knowledge, very few *E. cloacae* clonality studies are available. Recently, a multinational study performed on 195 rectal carriage isolates, revealed several epidemic CC, such as CC74 and CC114 (Izdebski et al., 2014). The STs observed in our study were reported by Izdebski et al. (2014) except for ST110. Our study confirms the findings of Izdebski et al. (2014) showing that some *E. cloacae* lineages have an increased epidemic potential and may participate to the spread of the resistance to antibiotics, including carbapenems (with or without carbapenemase genes).

The large clonal diversity of *E. cloacae* would be linked to antibiotics pressure. Further studies might point out specific factors (e.g., colonization, ability to infect more easily) explaining these findings.

## Conclusion

We combined here the molecular typing of carbapenem-non-susceptible *E. cloacae* isolates with the characterization of FQ resistance determinants. It will give new insights to the first multinational *E. cloacae* clonality study previously reported (Izdebski et al., 2014).

The MLST study revealed that *E. cloacae* comprised a large amount of lineages spreading in North-Eastern France. Nonetheless, *E. cloacae* ST74 was the most widespread ST, more often resistant to various antibiotics and carried more often PMQR. Carbapenemase production was not the major determinant leading to decrease susceptibility to carbapenem. Nonetheless carbapenemase-producing isolates carried the well-known IncL/M pOXA48a. In term of FQ-R, *E. cloacae* isolates harbored a wide range of pattern, from no substitution to multiple substitutions in QRDR. *qnr* was the major PMQR determinant found in *E. cloacae.*

These data underline, once again, the plausible role of the antibiotic pressure, in emergence and persistence of carbapenem and quinolones resistance genes.

## Conflict of Interest Statement

The authors declare that the research was conducted in the absence of any commercial or financial relationships that could be construed as a potential conflict of interest.

## Footnotes

1 http://www.infectiologie.com

2 www.EUCAST.org
