# Supplementary material for: Fluoroquinolone Resistance Mechanisms and population structure of Enterobacter cloacae non-susceptible to Ertapenem in North-Eastern France
Source: Front Microbiol. 2015 Oct 23;6:1186. doi: 10.3389/fmicb.2015.01186 (PMC4616961; doi:10.3389/fmicb.2015.01186)
Supplement: Supplementary file 1 [file Table_1.DOCX]

Table S1. List of primers used to map *bla*_OXA-48_ *in Tn1999.2.*

| Names | Sequence | Length (bp) | Tm (°C) | Reference |
| --- | --- | --- | --- | --- |
| Δtir_intR | TCCGGGCAGCTGATGTAATC | 20 | 59.9 | This study |
| TnpA1999_intF | AGCTGGTAAACCTGTACGCC | 20 | 60.0 | This study |
| IS1999B2INV | TCGTTTTAGGTGAAGTTCTGG | 21 | 55.8 | (Aubert et al., 2006) |
| blaOXA-48GSP2 | ACAGGCACAACTGAATATTTCATC | 24 | 57.8 | (Aubert et al., 2006) |
| TnpA1999_intF | AGCTGGTAAACCTGTACGCC | 20 | 60.0 | This study |
| Δtir3'ext | TCCTGTTCCGTGGTGTCATG | 20 | 60.0 | This study |
| OXA-48B_INV | GCCATCACAAAAGAAGTGCTC | 21 | 58.1 | (Aubert et al., 2006) |
| LysR3'ext | TCAGCGCCTTCTTGGACTTT | 20 | 59.9 | This study |
| LysRintF2_INV | TCGCACGCCTAGTTGTTCTT | 20 | 60.0 | This study |
| TnpA1999_intF2 | CCGTTCTTGATGCAGATGCG | 20 | 60.0 | This study |
| PemI3'ext | TCTGCCGAGATTTCAGCGTT | 20 | 60.0 | This study |
| rpoB-KpEcl_F | AAGGCGAATCCAGCTTGTTCAGC | 23 | 61.0 | (Doumith et al., 2009) |
| rpoB-KpEcl_R | TGACGTTGCATGTTCGCACCCATCA | 25 | 57.0 | (Doumith et al., 2009) |
| ompK35/ompF_F | TCCCTGCCCTGCTGGTAG | 18 | 55.0 | (Doumith et al., 2009) |
| ompF-Ecl_R2 | TAAGTGTTGTCGCCATCGTTG | 21 | 52.0 | (Doumith et al., 2009) |
| acrB-KpEcl_F | CGATAACCTGATGTACATGTCC | 23 | 53.0 | (Doumith et al., 2009) |
| acrB-KpEcl_R | CCGACAACCATCAGGAAGCT | 21 | 54.0 | (Doumith et al., 2009) |
